# Association Between Serum Advanced Glycation End Products and Cardiovascular‐Kidney‐Metabolic (CKM) Syndrome: A 3‐Year Longitudinal Cohort Study (2019–2022)

**DOI:** 10.1111/1753-0407.70137

**Published:** 2025-08-21

**Authors:** Hui Zhao, Ze‐wen Zhang, Tao Luo, Dilihumaer Aili, Wen‐huan Ding, Yuan‐yuan Li, Yuan‐sheng Gu, Shulipan Aslibek, Jing‐jing He, Wen‐hui Yu, Run‐ze Ma, Anaer Gaoshao, Ting‐ting Qiao, Guo‐zhen Zhang, Henry S. Lynn, Mu‐long Du, Jiang‐hong Dai

**Affiliations:** ^1^ Department of Epidemiology and Health Statistics School of Public Health, Xinjiang Medical University Urumqi China; ^2^ Key Laboratory of Special Environment and Health Research in Xinjiang Urumqi China; ^3^ Department of Epidemiology and Health Statistics School of Public Health, Institute of Medical Sciences of Xinjiang Medical University Urumqi China; ^4^ Central Laboratory of Xinjiang Medical University Urumqi China; ^5^ School of Nursing Xinjiang Medical University Urumqi China; ^6^ Department of Biostatistics Center for Global Health, School of Public Health, Nanjing Medical University Nanjing China; ^7^ Department of Urology The Second Affiliated Hospital with Nanjing Medical University Nanjing China

**Keywords:** cardiovascular‐kidney‐metabolic syndrome, cohort study, serum advanced glycation end products

## Abstract

**Background:**

Cardiovascular‐kidney‐metabolic (CKM) syndrome begins with obesity and glucose abnormalities, advancing to cardiovascular and kidney complications. This study investigates the relationship of advanced glycation end products (AGEs) with CKM syndrome staging and transition patterns.

**Methods:**

This 3‐year longitudinal study (2019–2022) of 1264 adults identified five CKM trajectory groups: Group 1 (stable low‐risk, 6.7%, stage 0/1), Group 2 (fluctuating, 15.8%, stages 0/1–2), Group 3 (stable intermediate, 52.8%, stage 2), Group 4 (progressors, 8.9%, to stage 3/4), and Group 5 (stable high‐risk, 15.8%, stage 3/4), from baseline distributions of stage 0 (1.6%), 1 (12.3%), 2 (71.0%), 3 (5.8%), and 4 (9.2%). Serum AGEs were quantified by UPLC‐MS/MS.

**Results:**

Higher AGEs levels showed significant associations with CKM severity, with each 1‐SD increase corresponding to a 30% greater likelihood of advanced staging (95% CI:10%–54%). Quartile analysis revealed a dose–response relationship (Q2:1.66[1.15–2.41]; Q3:1.67[1.12–2.48]; Q4:1.92[1.31–2.81]). Longitudinally, the total AGEs score was significantly associated with CKM transition patterns from 2019 to 2022. The odds ratios (ORs) for Group 2, Group 3, Group 4, and Group 5 compared to Group 1 were 1.61 (1.06–2.45), 1.64 (1.11–2.41), 1.71 (1.07–2.73), and 2.03 (1.32–3.13), respectively.

**Conclusions:**

These findings suggest that serum AGEs are linked to CKM severity and progression, potentially serving as biomarkers for CKM staging and targets for intervention.

## Graphical abstract

Our study reveals that elevated serum Advanced glycation end products (AGEs) are significantly associated with both the severity and progression of cardiovascular‐kidney‐metabolic (CKM) syndrome. The findings demonstrate dose‐dependent severity associations (adjusted OR = 1.92, Q4 vs. Q1) and predict 3‐year disease worsening (high‐risk vs. low‐risk: OR = 2.03, 95% CI 1.32–3.13), positioning AGEs as diagnostic biomarkers and modifiable therapeutic targets for this diabetes complication.

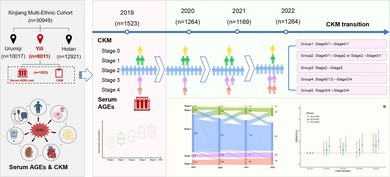

**Summary**

- Higher serum AGEs associate with advanced CKM stages (OR = 1.30, 95% CI 1.10–1.54), demonstrating a strong dose–response relationship (Q4 vs. Q1:OR = 1.92, 95% CI 1.31–2.81) in a cross‐sectional study.
- Longitudinal data (2019–2022) demonstrate baseline serum AGEs predict 3‐year CKM worsening (stable high‐risk [Group 5] vs. stable low‐risk [Group 1]: OR = 2.03, 95% CI 1.32–3.13).
- Serum AGEs may serve as biomarkers for CKM staging and potential targets for intervention.

## 1 Introduction

Cardiovascular‐kidney‐metabolic (CKM) syndrome is defined as a systemic disorder that arises from connections among obesity, diabetes, chronic kidney disease (CKD), and cardiovascular disease (CVD) [1]. CKM syndrome affects nearly all organ systems, resulting in premature mortality, excessive morbidity, and a particularly severe impact on the incidence of CVD [2, 3]. The American Heart Association (AHA) currently defines CKM syndrome through a staging system (stages 0–4), progressing from no risk factors (stage 0) to established cardiovascular disease (stage 4). This framework highlights CKM syndrome as a pathophysiologically interconnected multisystem disorder [4]. A 2024 NHANES analysis (2011–2020) revealed that ~90% of U.S. adults met CKM syndrome criteria (stage 1+), with 15% at advanced stages, showing no improvement over the decade [5]. Notably, China faces a similarly heavy CKM disease burden [3]. In recent years, some studies have reported on the relationships between neutrophil/high‐density lipoprotein cholesterol ratio [6], the systemic immune‐inflammation index [7], triglyceride glucose‐body mass index [3] and CKM syndrome. However, more prospective studies are needed to explore potential biomarkers or driving factors underlying the development of CKM syndrome and to develop more precise preventive strategies to slow disease progression.

Advanced glycosylation end products (AGEs) are irreversible compounds formed through the non‐enzymatic glycosylation of reducing sugars with nucleic acids, lipids, or proteins, which are heterogeneous and potentially harmful [8]. The binding of AGEs to their receptor, RAGE, induces insulin resistance, metabolic dysregulation, pancreatic β‐cell toxicity, and epigenetic modifications [9]. It is widely accepted that AGEs/RAGE play a central role in the occurrence and development of complications of diabetes [10]. However, research evidence on the association of AGEs with CKM syndrome is still limited. AGEs can be formed endogenously or supplied exogenously [11]. In addition, diet is the primary source of exogenous AGEs, and understanding the relationship between AGEs and CKM syndrome could provide new insights into dietary interventions to prevent the severe progression of CKM syndrome.

Therefore, in the current study, leveraging data from the Xinjiang Natural Population Cohort Study, initiated in 2019, we explored the relationship between AGEs and CKM syndrome and applied the 2022 follow‐up data to analyze the progression of CKM syndrome and AGEs, which will provide scientific evidence and dietary guidelines for the prevention and management of CKM syndrome.

## 2 Methods

### 2.1 Population and Design

As illustrated in Figure S1, the study participants were recruited from individuals enrolled in the Yili region of the Xinjiang Multi‐Ethnic Cohort (XMC). The XMC, initiated in 2018 and 2019, included 30 949 Chinese adults aged 35–74 years from three locations (Urumqi, Hotan and Yili) in Xinjiang, China, and conducted annual follow‐ups from 2020 to 2022. Detailed descriptions of the study design and data acquisition methods within the XMC have been published previously [12].

A total of 1523 participants with complete serum AGEs testing and CKM syndrome diagnostic data at baseline were included in this study. Among them, 1264 subjects had at least one follow‐up assessment between 2019 and 2022. CKM syndrome transitions were classified into five outcome patterns based on changes in CKM syndrome stage from 2019 to 2022: Stage0/1 → Stage0/1 (Group1), Stage0/1 → Stage2 or Stage2 → Stage0/1 (Group2), Stage2 → Stage2 (Group3), Stage0/1/2 → Stage3/4 (Group4) and Stage3/4 → Stage3/4 (Group5). The study was approved by the Ethics Committee of Xinjiang Medical University (XJYKDXR20220725015). All participants signed an informed consent document.

### 2.2 Collection and Definition

Sociodemographic, lifestyle, dietary, and medical history data were collected through face‐to‐face interviews using standardized questionnaires. The Dietary Inflammatory Index (DII) score was calculated as previously described by Shivappa et al. [13]. Anthropometric and biochemical measurements were obtained by trained staff following standardized protocols.

CKM syndrome staging was evaluated based on the AHA's CKM staging criteria (see Table S1) [1, 14, 15]. The estimated glomerular filtration rate (eGFR) was calculated using the Chronic Kidney Disease Epidemiology Collaboration (CKD‐EPI) creatinine equation [16]. Serum‐free AGEs, including carboxymethyllysine (CML), carboxyethyllysine (CEL), and methylglyoxal‐hydroimidazolone isomer (MG‐H1), were quantified using a modified ultra‐performance liquid chromatography–tandem mass spectrometry (UPLC‐MS/MS) method, as previously described with minor modifications [17]. Additional details are provided in the Data S1 (Tables S2–S4 and Figure S2).

### 2.3 Statistical Analysis

Data are presented as mean ± SD for normally distributed variables or median (IQR) for skewed distributions. Categorical variables are reported as frequencies (%). Group differences in CKM stages were analyzed using Kruskal‐Wallis tests. Due to skewed distributions, serum AGEs were log‐transformed and standardized (Z‐scores). An overall AGEs score was derived by averaging the Z‐scores of three AGEs. Differences across CKM stages and transition groups were visualized using box plots and analyzed by ANOVA. The associations between AGEs levels and CKM syndrome or CKM syndrome stage transitions were analyzed using ordinal logistic regression with restricted cubic splines.

We performed ordinal logistic regression to evaluate associations between AGEs (analyzed as continuous variables) and AGEs score (by quantiles) with CKM stages, reporting ORs (95% CIs) and *p*‐values. Analyses progressed from unadjusted to adjusted models: Model 1 included only AGEs measures; Model 2 additionally adjusted for demographics and lifestyle factors. Linear trends were tested using quantile medians as continuous variables.

In sensitivity analyses, we performed two complementary approaches: [1] ordinal logistic regression after combining CKM stages (0–1 and 3–4), and [2] linear regression treating CKM stages as a continuous variable. For both approaches, Model 1 examined unadjusted associations with AGEs, while Model 2 adjusted for key covariates. All other parameter inclusion criteria were consistent with those of the main model.

A multinomial logistic regression model was used to analyze the relationships between AGEs scores (and each specific AGE) and transitions across different CKM syndrome stages over time (2019–2020, 2019–2021, and 2019–2022), with Group 1 as the reference group. Models for AGEs and CKM syndrome transition groups at different time periods were adjusted separately for demographic characteristics.

The threshold for statistical significance was set at *p* < 0.05 and two‐sided. Statistical analyses were performed using R software (version 4.4.0).

## 3 Results

### 3.1 Characteristics of the Participants

A total of 1523 participants were included at baseline, with 25 (1.64%), 188 (12.34%), 1082 (71.04%), 88 (5.78%), and 140 (9.19%) participants in stages 0 through 4, respectively. In 2022, 1264 participants were followed up, with 85 (6.72%) in Stage 0/1 → Stage 0/1 (Group 1), 200 (15.82%) in Stage 0/1 → Stage 2 or Stage 2 → Stage 0/1 (Group 2), 667 (52.77%) in Stage 2 → Stage 2 (Group 3), 112 (8.86%) in Stage 0/1/2 → Stage 3/4 (Group 4), and 200 (15.82%) in Stage 3/4 → Stage 3/4 (Group 5) (Tables S5, S6 and Figure S1).

The demographic characteristics of the participants are summarized in Table S5. Statistically significant differences were observed among the CKM syndrome staging groups in terms of education level, alcohol consumption, physical activity, as well as in age, DII, BMI, waist circumference, fasting glucose, triglycerides, HDL‐C, eGFR, and the prevalence of diabetes, hypertension, hypertriglyceridemia, high‐density lipoprotein abnormalities, and metabolic syndrome. Additionally, significant differences were noted in the median serum concentrations of CML, CEL, MG‐H1, and the overall AGEs score at baseline.

Statistically significant differences were observed in physical activity, as well as age, DII, BMI, waist circumference, fasting glucose, triglycerides, HDL‐C, eGFR, and the prevalence of diabetes, hypertension, hypertriglyceridemia, and metabolic syndrome. Additionally, significant differences were noted in the median serum concentrations of CML, CEL, MG‐H1, and the overall AGEs score among the CKM syndrome transition groups (Table S6).

### 3.2 The Relationship Between Serum AGEs and CKM Syndrome

**FIGURE 1:**
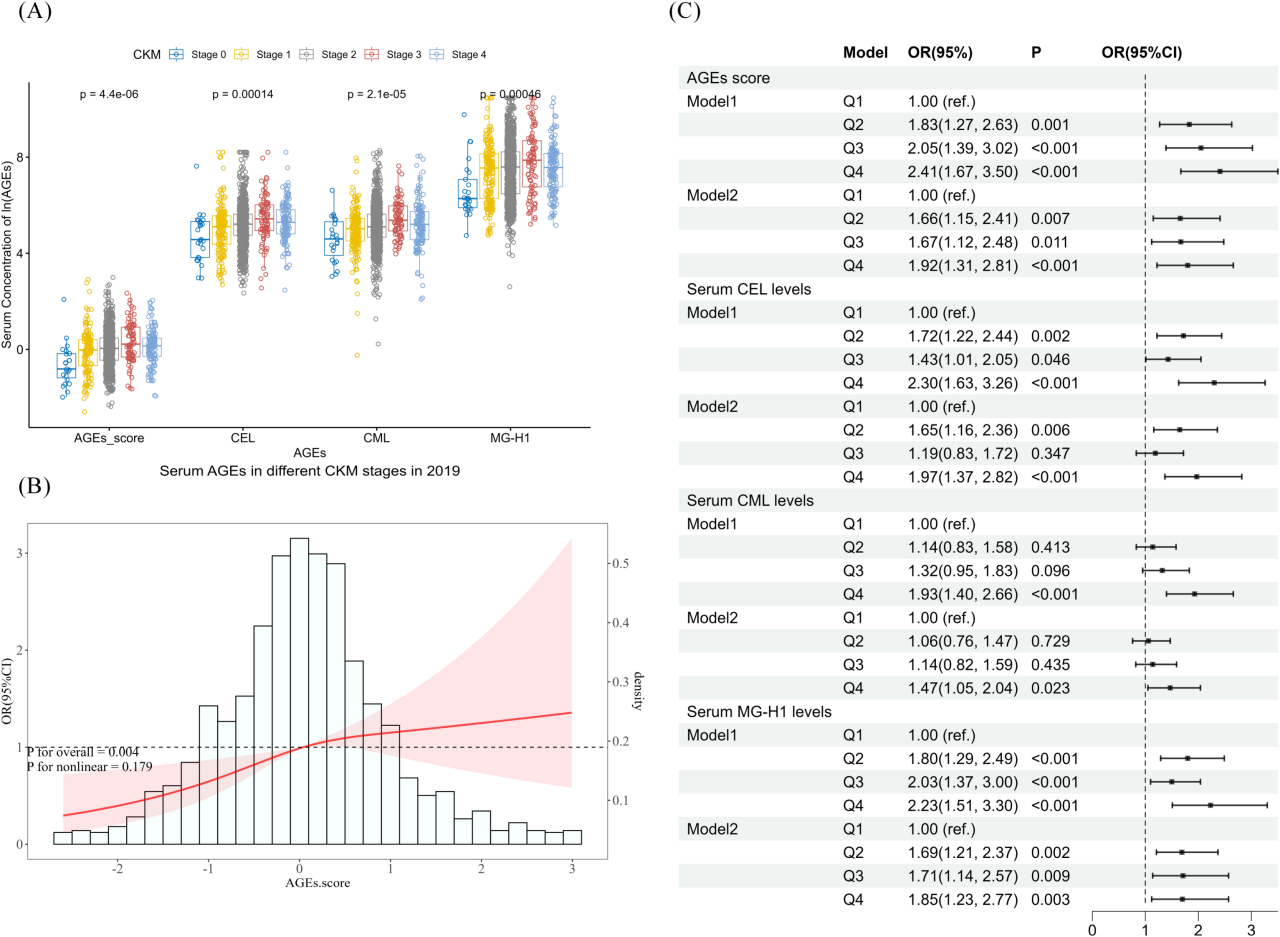
The relationship between serum AGEs and CKM syndrome. (A) Differences in AGEs among CKM syndrome stage subjects. (B) Linear/nonlinear relationship between AGEs score and CKM syndrome. (C) Association of AGEs score and concentration of specific serum AGEs with CKM syndrome in baseline. Serum AGEs concentration were adjusted for age, educational level, alcohol consumption, physical activity and DII in (B). Data are ORs (95% CIs) in (C). OR odds ratio, CI confidence interval. In (C) Model 1 Only AGEs score or concentration of specific serum AGEs. Model 2: adjusted for age, educational level, alcohol consumption, physical activity and dietary inflammation index. Q1 Quantile 1, Q2 Quantile 2, Q3 Quantile 3, Q4 Quantile 4.

The AGEs score and log‐transformed serum concentrations of CEL, CML, and MG‐H1 were found to be statistically different across CKM syndrome stages (Figure 1A). Restricted cubic spline analysis revealed a linear relationship between AGEs scores and the risk of CKM syndrome (*p* < 0.05), indicating that higher CKM syndrome stages were associated with elevated serum AGEs scores (Figure 1B). Similar trends were observed for each specific serum AGE (Figure S3).

Ordinal logistic regression was used to examine the relationship between CKM syndrome stages and serum AGEs scores, adjusting for demographic characteristics that were statistically different across CKM syndrome stages. A progressive increase in CKM syndrome stage was observed with higher AGEs scores. After adjusting for confounders, multivariate ordinal logistic regression analysis indicated that higher AGEs scores were independently associated with an increased risk of CKM syndrome stage progression.

Compared to the lowest quartile (Q1) of AGEs scores, the odds of having a CKM stage at least one grade higher were 1.66(95% CI: 1.15–2.41), 1.67 (95% CI: 1.12–2.48), and 1.92 (95% CI: 1.31–2.81) for Q2, Q3, and Q4, respectively. Higher AGEs scores were significantly associated with more advanced CKM stages, with each 1‐SD increase corresponding to a 30% (95% CI: 10%–54%) greater likelihood of being at a higher stage. The results for serum CEL, CML, and MG‐H1 concentrations, as well as the linear trend tests for AGEs scores using the median of each quartile, were generally consistent (Figure 1C and Table 1).

**TABLE 1:** Association of AGEs score and concentration of specific serum AGEs with CKM at the baseline.

CKM stage	Q1	Q2	Q3	Q4	P‐trend	1‐SD increment
	(−2.6, −0.45)	(−0.45, 0.0428)	(0.0428, 0.484)	(0.484, 2.99)		
CKM stage0/1/2/3/4, *n*						
Serum AGEs score levels						
Model 1	1.00 (ref.)	1.83 (1.27, 2.63)	2.05 (1.39, 3.02)	2.41 (1.67, 3.50)	< 0.001	1.42 (1.21, 1.67)
Model 2	1.00 (ref.)	1.66 (1.15, 2.41)	1.67 (1.12, 2.48)	1.92 (1.31, 2.81)	0.001	1.30 (1.10, 1.54)
Serum CEL levels						
Model 1	1.00 (ref.)	1.72 (1.22, 2.44)	1.43 (1.01, 2.05)	2.30 (1.63, 3.26)	< 0.001	1.31 (1.14, 1.50)
Model 2	1.00 (ref.)	1.65 (1.16, 2.36)	1.19 (0.83, 1.72)	1.97 (1.37, 2.82)	< 0.001	1.25 (1.08, 1.44)
Serum CML levels						
Model 1	1.00 (ref.)	1.14 (0.83, 1.58)	1.32 (0.95, 1.83)	1.93 (1.40, 2.66)	< 0.001	1.32 (1.17, 1.50)
Model 2	1.00 (ref.)	1.06 (0.76, 1.47)	1.14 (0.82, 1.59)	1.47 (1.05, 2.04)	0.020	1.20 (1.05, 1.36)
Serum MG‐H1 levels						
Model 1	1.00 (ref.)	1.80 (1.29, 2.49)	2.03 (1.37, 3.00)	2.23 (1.51, 3.30)	< 0.001	1.24 (1.11, 1.39)
Model 2	1.00 (ref.)	1.69 (1.21, 2.37)	1.71 (1.14, 2.57)	1.85 (1.23, 2.77)	0.006	1.16 (1.03, 1.31)

*Note:* Ordinal logistic regression was used to analyze the relationship between the different stages of CKM and the four quartiles of AGEs, and the strength of association was demonstrated by OR (95% CIs). Model 1: Only AGEs score or concentration of specific serum AGEs. Model 2: adjusted for age, educational level, alcohol consumption, physical activity, and dietary inflammation index. P‐trend: Linear trend test using the median value of each category. Q1, Quantile 1; Q2, Quantile 2; Q3, Quantile 3; Q4, Quantile 4.

Abbreviations: CI, confidence interval; OR, odds ratio.

In the sensitivity analysis, ordinal logistic regression models were constructed to examine the relationship between AGEs and CKM syndrome by combining stages 0 and 1 into one group and stages 3 and 4 into another group. The results were consistent with those of the main analysis (Table S7). In a second sensitivity analysis, CKM syndrome stages 0 through 4 were treated as a continuous variable in the model, and the results remained consistent with the main findings (Table S8).

### 3.3 The Relationship Between Serum AGEs and CKM Syndrome Transition

As shown in Figure S4, AGEs scores, and serum CEL, CML, and MG‐H1 concentrations were statistically different across the CKM syndrome stage transition groups over the three‐year follow‐up period (2019–2022). After adjusting for confounders, AGEs scores, and serum CML, CEL, and MG‐H1 concentrations exhibited a positive linear trend with CKM syndrome stage changes over the three‐year period (2019–2022), suggesting that higher serum AGEs scores are associated with more severe CKM syndrome stage transitions (Figure S5).

**FIGURE 2:**
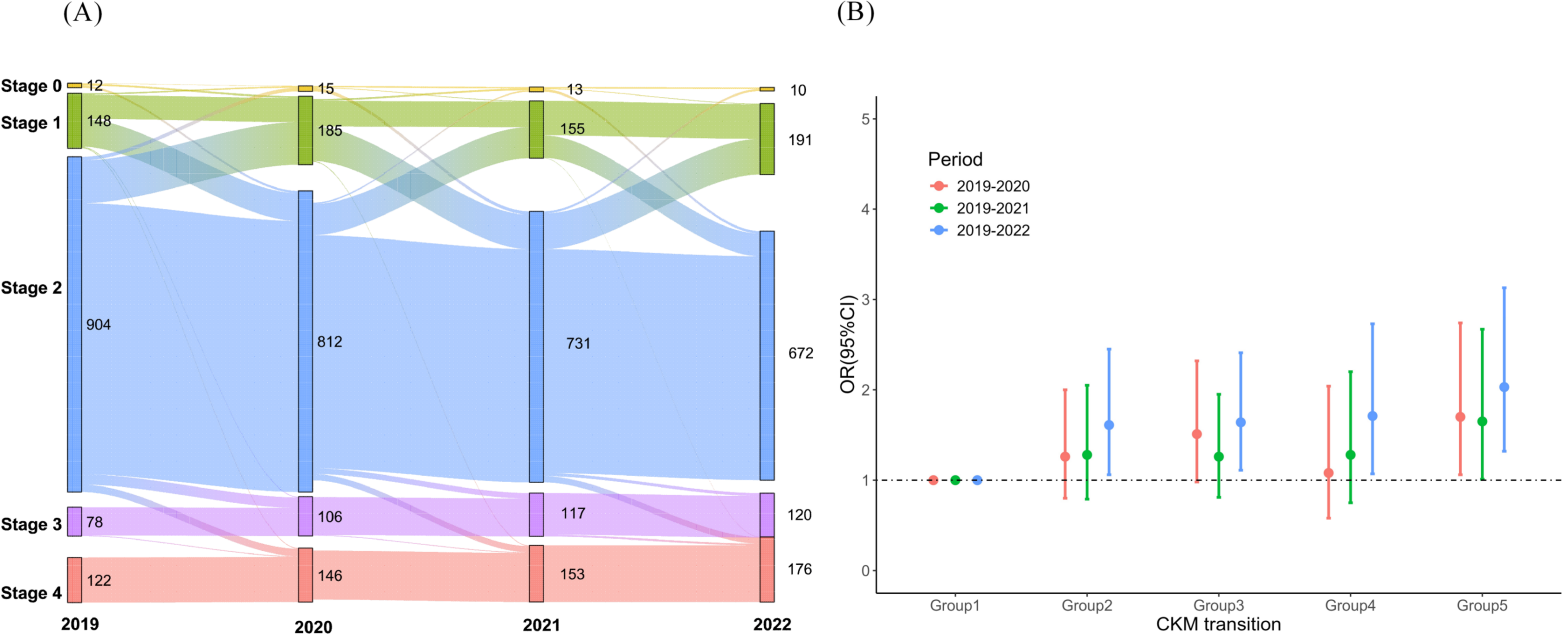
The relationship between serum AGEs and CKM syndrome stage transitions. (A) The transition of CKM syndrome stage from 2019 to 2022. (B) Relationship between AGEs scores and CKM syndrome stage transitions at different time periods. Data are ORs (95% CIs). OR, odds ratio for the association per 1‐SD increment of standard; CI confidence interval. 2019–2020 period: adjusted for age, education level, physical activity, smoking status, and dietary inflammation index. 2019–2021 period: adjusted for age, education level, smoking status, physical activity; 2019–2022 period: adjusted for age, education level, and physical activity; Group1: Stage0/1 → Stage0/1; Group2: Stage0/1 → Stage2 or Stage2 → Stage0/1; Group3: Stage2 → Stage2; Group4: Stage0/1/2 → Stage3/4; Group5: Stage3/4 → Stage3/4.

In the multinomial logistic regression model, after adjusting for demographic characteristics that showed statistically significant differences across CKM syndrome stages, each 1‐standard deviation (SD) increase in AGEs scores was associated with significantly higher odds of specific stage transitions: 1.61 (95% CI: 1.06–2.45) times higher odds of transitioning from Stage 0/1 to Stage 2 or from Stage 2 to Stage 0/1, 1.64 (95% CI: 1.11–2.41) times higher odds of remaining at Stage 2 (Stage 2 → Stage 2), 1.71 (95% CI: 1.07–2.73) times higher odds of progressing to Stage 3/4 from any lower stage (Stage 0, 1, or 2), and 2.03 (95% CI: 1.32–3.13) times higher odds of remaining at Stage 3/4 (Stage 3/4 → Stage 3/4), using the group that remained at Stage 0/1 (Stage 0/1 → Stage 0/1) as the reference. The results were consistent across the 2019–2020 and 2019–2021 time periods (Table 2 and Figure 2).

**TABLE 2:** Relationship between AGEs scores and CKM syndrome stage transitions at different time periods.

CKM syndrome stage transitions	2019–2020		2019–2021		2019–2022	
	OR (95% CI)	*p*‐value	OR (95% CI)	*p*‐value	OR (95% CI)	*p*‐value
Stage0/1 → Stage0/1 (*n* = 85)	ref.		ref.		ref.	
Stage0/1 → Stage2 or Stage2 → Stage0/1 (*n* = 200)	1.26 (0.80, 2.00)	0.317	1.28 (0.79, 2.05)	0.312	1.61 (1.06, 2.45)	0.02
Stage2 → Stage2 (*n* = 667)	1.51 (0.98, 2.32)	0.06	1.26 (0.81, 1.95)	0.303	1.64 (1.11, 2.41)	0.012
Stage0/1/2 → Stage3/4 (*n* = 112)	1.08 (0.58, 2.04)	0.804	1.28 (0.75, 2.2)	0.365	1.71 (1.07, 2.73)	0.025
Stage3/4 → Stage3/4 (*n* = 200)	1.70 (1.06, 2.74)	0.028	1.65 (1.01, 2.67)	0.043	2.03 (1.32, 3.13)	0.001

*Note:* 2019–2020 period: adjusted for age, education level, physical activity, smoking status, and dietary inflammation index. 2019–2021 period: adjusted for age, education level, smoking status, physical activity; 2019–2022 period: adjusted for age, education level, and physical activity.

## 4 Discussion

In this study, we first investigated the relationship between serum AGEs levels and CKM syndrome stages through cross‐sectional analysis and observed that AGE score, CEL, CML, and MG‐H1 were positively associated with the severity of CKM syndrome stages. Furthermore, our longitudinal cohort study with a three‐year follow‐up period substantiated the impact of baseline serum AGEs levels on CKM syndrome progression. These findings provide compelling evidence that higher baseline AGEs levels may play a contributory role in the pathogenesis and progression of CKM syndrome.

Growing evidence establishes CKM syndrome as a systemic disorder driven by the pathophysiological interplay of obesity, diabetes, CVD, and CKD [18, 19]. This multisystem convergence poses a major public health challenge, particularly in industrialized nations, where it accounts for a substantial morbidity–mortality burden [20]. A 2024 JAMA study (2011–2020) found widespread poor CKM health among U.S. adults, highlighting its urgent recognition as a public health priority 5 [5]. In China, specific data on CKM syndrome are currently limited. This study revealed that the proportions of stage 3 and stage 4 CKM in the Chinese population were 5.78% and 9.19%, respectively. These figures align closely with the prevalence observed in U.S. adults from 2011 to 2020, where stage 3 and stage 4 CKM prevalence were reported as 5.4% and 9.2%, respectively [5].

The progression of CKM to stage 3 and stage 4 indicates that patients have developed irreversible chronic kidney disease or cardiovascular disease. Further research is required to clarify CKM progression pathways, enabling the development of precision interventions to halt advancement to late‐stage disease. The study on CKM prevalence in the U.S. is based on NHANES, which relies on multiple waves of cross‐sectional data. This design lacks longitudinal progression data from the same cohort, thereby limiting the ability to analyze factors influencing CKM development and outcomes. In contrast, our study, which included a three‐year follow‐up, revealed that 15.82% of individuals experienced unstable metabolic status (transitioning between Stage 0/1 and Stage 2) and 8.86% exhibited worsening CKM (progressing from Stage 0/1/2 to Stage 3/4). These findings suggest significant potential for progression to advanced stages (Stage 3 or Stage 4) of CKM syndrome. Consequently, greater attention should be directed toward early screening and intervention across all stages of CKM to mitigate disease progression and improve outcomes.

The staging of CKM syndrome outlines a progressive continuum, beginning with the absence of risk factors (Stage 0), advancing to overweight/obesity or impaired fasting glucose (Stage 1), and further progressing to metabolic abnormalities such as hypertension, hyperlipidemia, or type 2 diabetes (Stage 2). This continuum ultimately culminates in the development of clinically diagnosed CVD or CKD (Stage 3 and Stage 4). This framework clearly demonstrates that CKM is a continuously progressive systemic syndrome. Historically, due to the lack of a systematic understanding and definition of CKM syndrome, research has often focused on individual diseases or components, such as obesity, type 2 diabetes, CVD, or CKD. However, such fragmented studies fail to fully capture the cumulative health impacts of these interconnected conditions and cannot propose comprehensive prevention and control strategies. Moving forward, it is essential to re‐examine CKM as a unified systemic disease and identify biomarkers that reflect its progression, enabling a more holistic approach to its management and intervention. Several studies have reported associations between AGEs and diabetes, as well as its complications, including CVD and CKD [21]. The results of this study demonstrated a significant association between increased CKM syndrome stages and elevated serum AGE levels, suggesting a potential association between serum AGEs and both the risk factors and the severity of CKM syndrome.

Several underlying mechanisms have been proposed to explain the association between CKM syndrome stages and serum AGEs levels. Throughout the progression of CKM syndrome, the presence of overweight/obesity and hyperglycemia (at stage 1) may significantly contribute to the emergence of pathophysiological conditions including metabolic syndrome, chronic kidney disease, and cardiovascular disease. This contribution is mediated through a variety of biological pathways, notably inflammation via the AGEs‐RAGE pathway, oxidative stress, insulin resistance, and vascular dysfunction [20, 22, 23, 24]. In addition, the binding of AGEs to RAGE induces the formation of reactive oxygen species (ROS) and activates signaling proteins such as extracellular signal‐regulated kinase 1/2 (ERK1/2). This activation subsequently increases the expression and phosphorylation of nuclear factor κ‐light‐chain enhancer of activated B cells (NF‐κB), leading to oxidative stress and inflammation. These processes contribute to pathological changes, including left ventricular remodeling, further exacerbating cardiovascular complications [25]. In addition, AGEs and RAGE have been found to promote atrial fibrillation in animal studies because they are involved in the remodeling of atrial structure in diabetic rats [26]. The AGE‐RAGE pathway plays a role in the initiation and progression of coronary artery disease (CAD) and myocardial infarction (MI) and in cardiac remodeling occurring after acute events [27]. Individuals with diabetes, obesity, and metabolic syndrome are at increased risk of developing CKD [28]. The AGEs‐RAGE pathway also mediates the development of nephropathy [29].

The study demonstrated that individuals with elevated baseline levels of AGEs were more likely to develop clinically diagnosed CKD and CAD within a three‐year period. These findings suggest that reducing serum AGEs levels may help alleviate metabolic disturbances and prevent the progression of CKM syndrome to advanced stages (Stage 3 or Stage 4). Supporting this notion, a Japanese study found that patients with type 2 diabetes who maintained consistently low serum MG‐H1 levels had a reduced risk of cardiovascular events [30]. These collective results highlight the potential of targeting AGEs reduction as a novel strategy to slow the progression of CKM syndrome and mitigate its associated complications.

Several strengths of this study should be acknowledged. To the best of our knowledge, this is the first prospective cohort study to investigate the relationship between serum AGEs and CKM syndrome in adults from Xinjiang, China. The study cohort comprises a large, multi‐ethnic natural population, which enhances the generalizability and applicability of the findings. Secondly, the follow‐up study, conducted from 2019 to 2022, provided annual CKM syndrome status updates for this multi‐ethnic cohort, enabling a robust exploration of the relationship between AGEs and CKM syndrome progression. This longitudinal design offers stronger evidence that reducing AGEs levels may help mitigate the progression of CKM syndrome. Thirdly, while various methods have been used to detect serum AGEs in previous studies, this study utilized ultra‐performance liquid chromatography–mass spectrometry (UPLC‐MS/MS), which provides the most accurate quantification of AGEs. Finally, the study employed multiple sensitivity analysis approaches, including the inclusion of multiple covariates, the application of various regression models, and modeling across different time periods, to ensure the robustness and reliability of the results.

However, certain limitations should be acknowledged when interpreting our findings. First, the diagnosis of established CVD relied on self‐reported data due to unavailable objective diagnostic confirmation (e.g., imaging or biomarker verification). This methodological limitation may have resulted in the underestimation or overestimation of the prevalence of stage 3 and 4 CKM syndrome. Second, CKD staging was based exclusively on estimated glomerular filtration rate (eGFR) thresholds owing to unavailable albuminuria data. Although eGFR is a validated renal function metric, this approach may underestimate true CKD prevalence. Future validation studies should incorporate albuminuria measurements to improve CKM staging accuracy. Third, the risk factors and pathogenic mechanisms underlying CKM syndrome are highly complex. Although we controlled for potential confounders in multivariate analyses, the possibility of residual confounding from unmeasured factors cannot be entirely excluded. Finally, this study utilized a single‐center design with a regionally specific cohort (Xinjiang, China), and all participants were Chinese adults. While this approach enhances internal consistency and contextual relevance to the target population, it may limit the generalizability of the findings to other populations with different genetic, environmental, or lifestyle backgrounds.

## 5 Conclusions

This study provides evidence that serum AGEs levels were positively correlated with both the stage and transition of CKM syndrome, suggesting that AGEs may serve as valuable biomarkers for assessing disease severity and as potential therapeutic targets for interventions. These findings could aid in the development of targeted strategies to prevent the onset and progression of CKM syndrome; ultimately improving clinical outcomes.

## Author Contributions

H.Z., J.H.D., and M.L.D. conceptualized the study; H.Z., W.H.D., Z.W.Z., and T.T.Q. developed the methodology and conducted the analysis; H.Z., Z.W.Z., T.L., D.A., Y.Y.L., Y.S.G., L.P.S., J.J.H., W.H.Y., R.Z.M., A.G., and G.Z.Z. carried out the investigation; J.H.D. provided resources; H.Z., Z.W.Z., and T.L. curated the data; H.Z. wrote the main manuscript; H.Z., J.H.D., H.S.L., and M.L.D. reviewed and edited the manuscript; H.Z. created the visualizations; J.H.D. supervised the project; J.H.D. managed project administration; H.Z. and J.H.D. acquired funding. All authors have read and agreed to the published version of the manuscript.

## Ethics Statement

The study was conducted in accordance with the Declaration of Helsinki and received ethical approval from both the Ethics Committee of the Xinjiang Uygur Autonomous Region Academy of Traditional Chinese Medicine (Approval No. 2018XE0108) and the Ethics Committee of Xinjiang Medical University (Approval No. XJYKDXR20220725015).

## Consent

Written informed consent has been obtained from the subjects to publish this paper.

## Conflicts of Interest

The authors declare no conflicts of interest.

## Supporting information


**Data S1:** Suppotimg Information.

**Data S2:** Suppotimg Information.

## Data Availability

Data available on request due to restrictions for exampe, privacy or ethical.
