# Supplementary material for: Association Between Serum Advanced Glycation End Products and Cardiovascular‐Kidney‐Metabolic (CKM) Syndrome: A 3‐Year Longitudinal Cohort Study (2019–2022)
Source: J Diabetes. 2025 Aug 21;17(8):e70137. doi: 10.1111/1753-0407.70137 (PMC12370401; doi:10.1111/1753-0407.70137)
Supplement: Supplementary file 1 — Data S1: Suppotimg Information. [file JDB-17-e70137-s001.docx]

**Supplements**

**Contents**

Methods S1. Variable Definitions and Measurement

Methods S2. Quantification of Advanced Glycation End-products Using UPLC-MS

Figure S1. Flowchart of select subjects process

Figure S2. Specificity of AGEs in human serum samples

Figure S3. Linear/nonlinear relationship between AGEs score and concentration of specific serum AGEs and CKM at baseline

Figure S4. Differences in AGEs in different transition groups of CKM in different periods

Figure S5. Linear/nonlinear relationship between AGEs score/concentration of specific serum AGEs and different transition groups of CKM in 2019-2022

Table S1. The definitions of CKM syndrome

Table S2. LC-MS/MS experimental parameters setting

Table S3. Calibration standard concentration curves for each serum AGEs concentration

Table S4. Precision, accuracy, recovery for the determination of AGEs

Table S5. Demographics and clinical characteristics of subjects at the baseline (N = 1523)

Table S6. Demographic and clinical characteristics of subjects in the CKM stage transition group, 2019-2022

Table S7. Sensitivity analysis for relationship between AGEs scores or specific serum AGEs concentrations and CKM at the baseline by combining CKM's stage0 and stage1, stage 3 and stage 4

Table S8. Sensitivity analysis for relationship between AGEs scores and serum specific AGEs concentration with CKM at the baseline by CKM as continuous variable

**Methods S1.** **Variable Definitions and Measurement**

Sociodemographic characteristics (age, sex, and education), lifestyle factors (smoking, tea consumption, alcohol use and physical activity patterns) and dietary were collected by through face-to-face interviews using standardized questionnaires.

Medical history (including hypertension treatment medications and statin use) was collected via face-to-face interviews conducted by trained primary healthcare providers (i.e., village doctors). These interviewers possessed professional training and had longitudinal familiarity with the participants' general health status over recent years. This approach was implemented to mitigate recall bias and interviewer bias to a certain extent.

Educational attainment was classified as primary school or below versus junior high school or above. Smoking, alcohol consumption, and tea consumption were categorized as never or present/former. Physical activity patterns were classified as light or moderate/heavy. Individual dietary data were collected using a semi-quantitative food frequency questionnaire (FFQ). The Dietary Inflammatory Index (DII) score was calculated as previously described by Shivappa et al ^13.^ Height, weight, waist circumference, blood pressure, lipid indices, fasting blood glucose (FBG) and serum creatinine (SCr) were measured during physical examinations and blood biochemical tests, which were conducted by trained physicians or nurses following standardized protocols.

According to the American Heart Association’s CKM syndrome staging criteria, overweight/obesity was defined as BMI ≥ 23 kg/m², abdominal obesity as waist circumference ≥ 90 cm (men) or ≥ 85 cm (women), hypertension as SBP/DBP ≥ 130/80 mmHg or self-reported history, hypertriglyceridemia as TG-C ≥ 1.525 mmol/L, abnormal HDL-C as ≤ 1.30 mmol/L (women) or ≤ 1.04 mmol/L (men), hyperglycemia as FBG ≥ 5.6 mmol/L (with 5.6-6.9 mmol/L indicating adipose dysfunction), and diabetes as FBG ≥ 7.0 mmol/L or self-reported history, metabolic syndrome (MetS) required ≥ 3 of these abnormalities.

The estimated glomerular filtration rate (eGFR), a measure of renal function, was calculated using the Chronic Kidney Disease Epidemiology Collaboration (CKD-EPI) creatinine equation ^16^. The CKD-EPI formula incorporates age, sex, and serum creatinine levels, and its calculation was implemented using the “transplantr” R package. Chronic kidney disease (CKD) is staged according to eGFR values as follows: CKD stage 1, eGFR > 90ml·min-1·(1.73m^2^)-1; CKD stage 2, eGFR 60-89 ml·min-1·(1.73m^2^)-1; CKD stage 3a, eGFR 45-59 ml·min-1·(1.73m^2^)-1; CKD stage 3b, eGFR 30-44 ml·min-1·(1.73m^2^)-1; CKD4 stage, eGFR 15-29 ml·min-1·(1.73m^2^)-1; CKD5 stage, eGFR < 15 ml·min-1·(1.73m^2^)-1.

Cardiovascular disease (CVD) includes coronary heart disease, heart failure, stroke, peripheral artery disease and atrial fibrillation. Subclinical cardiovascular disease was assessed based on a high predicted 10-year risk of CVD, calculated using the American Heart Association (AHA) Predicting Risk of Cardiovascular Disease Events (PREVENT) equation 15 The 10-year risk for CVD is categorized as follows: low risk (<5%), borderline risk (5% to 7.4%), intermediate risk (7.5% to 19.9%), and high risk (≥20%) 16. The definitions of CKM syndrome as the Table S1:

**Methods S2. Quantification of Advanced Glycation End-products Using UPLC-MS**

Participants with hematological disorders were excluded and asked to rest before blood collection. Fasting venous blood (5 mL) was drawn, centrifuged at 3000 rpm for 10 minutes (4°C), and serum was stored at -80°C. Serum-free advanced glycation end products (AGEs), including carboxymethyllysine (CML), carboxyethyllysine (CEL), and methylglyoxal-hydroimidazolone isomer (MG-H1), were quantified using a modified ultra-performance liquid chromatography-tandem mass spectrometry (UPLC-MS/MS) method, as previously described with minor modifications ^17^. All analyses were performed at the Central Laboratory of Xinjiang Medical University. In brief, 100 μL of serum was vortex-mixed with 25 μL of the internal standard CML-d4 (1 μg/mL) in a 2 mL EP tube. A 400 μL methanol-acetonitrile mixture (1:3 ratio) was added to precipitate proteins. After centrifugation at 15,000 rpm for 20 minutes, the supernatant was collected and dried under nitrogen stream at 60°C at low speed until completely dry. The residue was then re-solubilized in 200 μL of 2 mmol/L aqueous perfluoropentanoic acid, vortexed to ensure complete dissolution, filtered through a membrane, and transferred to an injection vial for UPLC-MS/MS analysis.

Two microliters of the prepared solution were injected into the UPLC-MS/MS system. Liquid chromatography was performed at 40°C using an Acquity UPLC BEH C18 column (1.7 μm, 2.1 × 100 mm; Waters, Milford, MA, USA). The Micromass Quattro Premier XE Tandem Mass Spectrometer (Waters) was operated in multiple reaction monitoring (MRM) mode with electrospray ionization (ESI) in positive ion mode. The intra-assay coefficients of variation (CVs) for CML, CEL, and MG-H1 assays were 2.45%, 1.81%, and 4.61%, respectively. The inter-assay CVs were 5.45%, 9.58%, and 7.27%, respectively. Additional details are provided in the Supplementary Data (Tables S2–S4 and Figure S2).


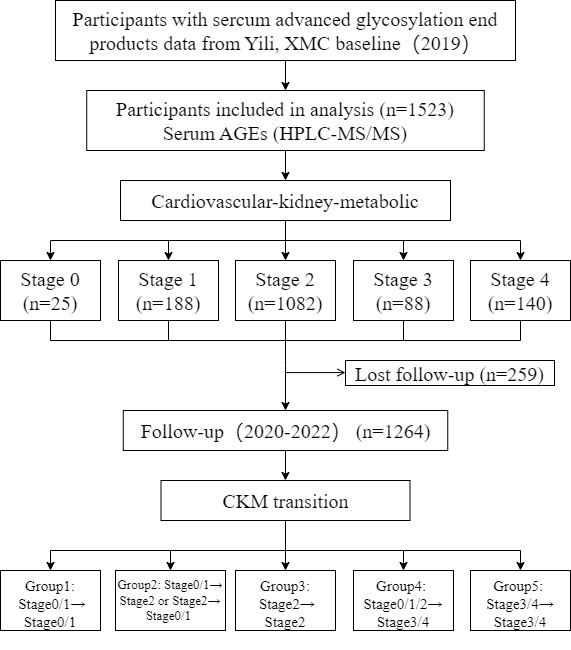


**Figure S1. Flowchart of select subjects process.**

|  |  |
| --- | --- |

**Figure S2. Specificity of AGEs in human serum samples**

| 1. CEL   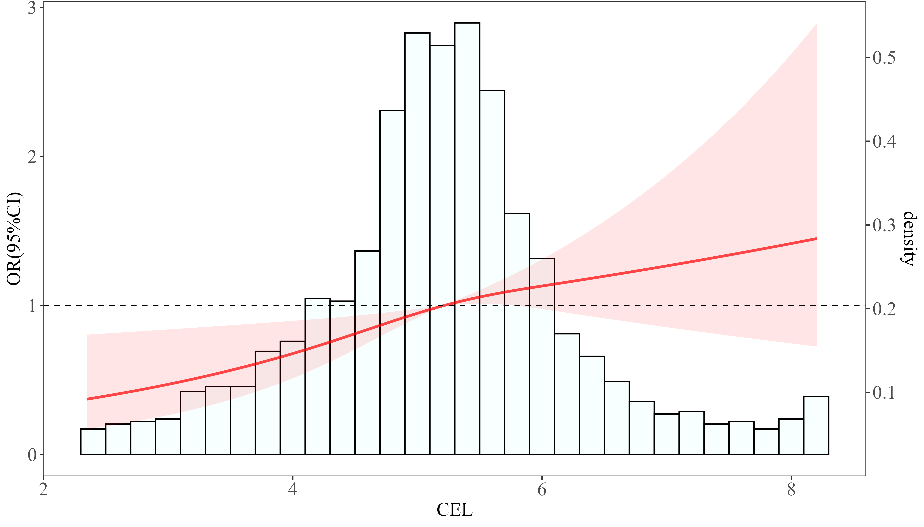 | 1. CML   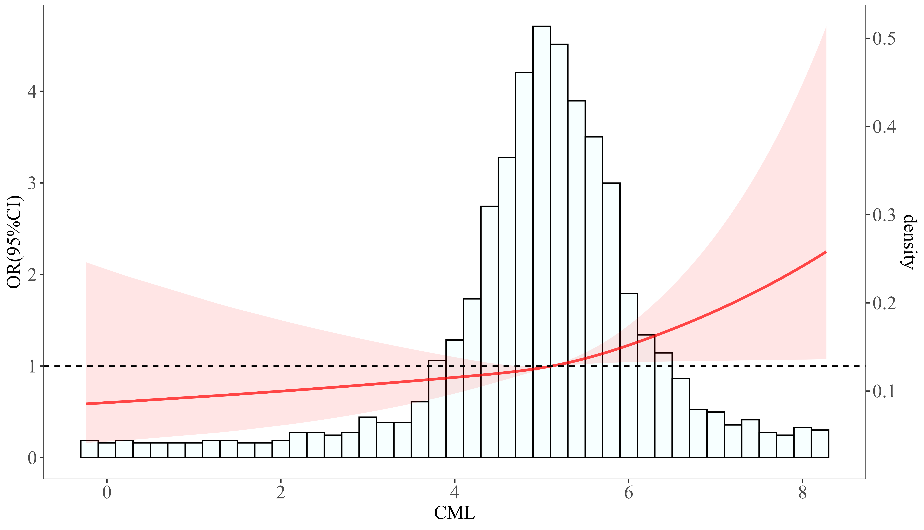 |
| --- | --- |
| 1. MG-H1   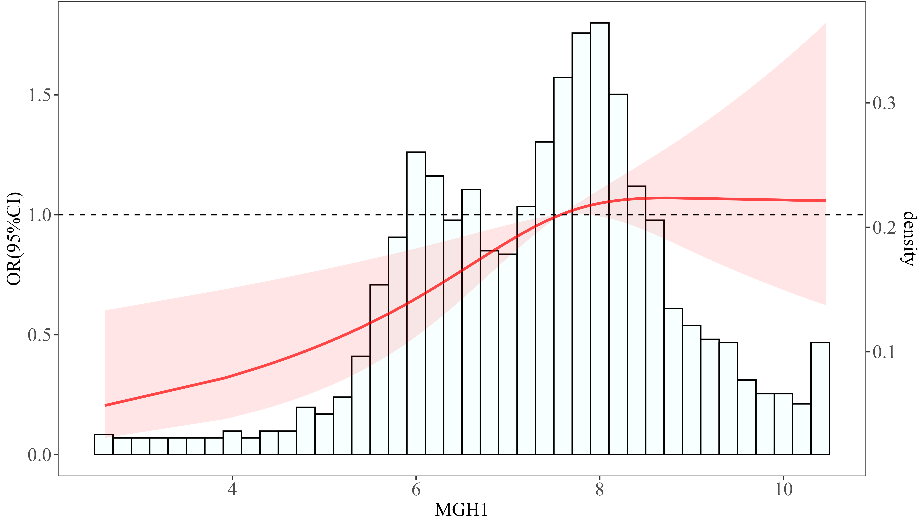 |  |

**Figure S3.** **Linear/nonlinear relationship between AGEs score and concentration of specific serum AGEs and CKM at baseline**

Note: The restricted cubic splines were using to analysis the relationship between AGEs score and concentration of specific serum AGEs and CKM, and the analyses were adjusted for age, educational level, alcohol consumption, physical activity and dietary inflammation index. (A) Carboxyethyllysine (CEL), (B) Carboxymethyllysine (CML), and (C) Methylglyoxyl-hydroimidazolone-1 (MG-H1).

| 1. 2019-2020   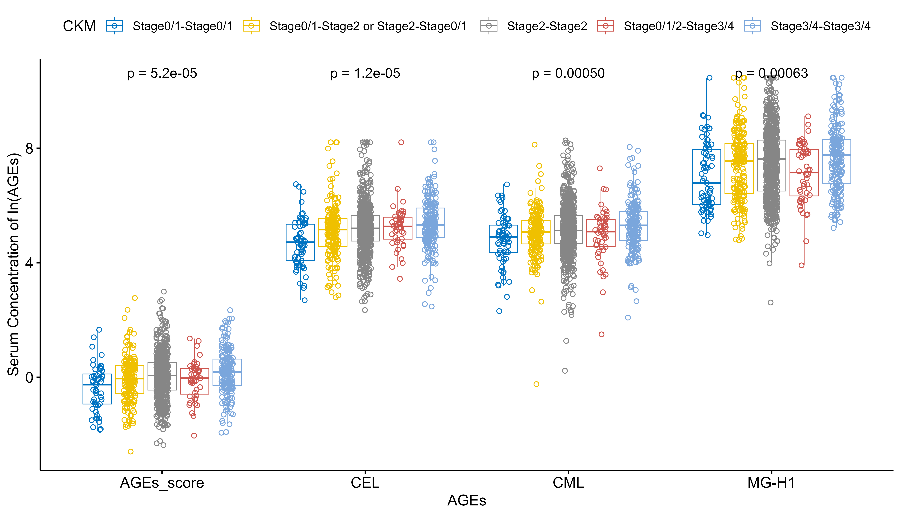 |  | 1. 2019-2021   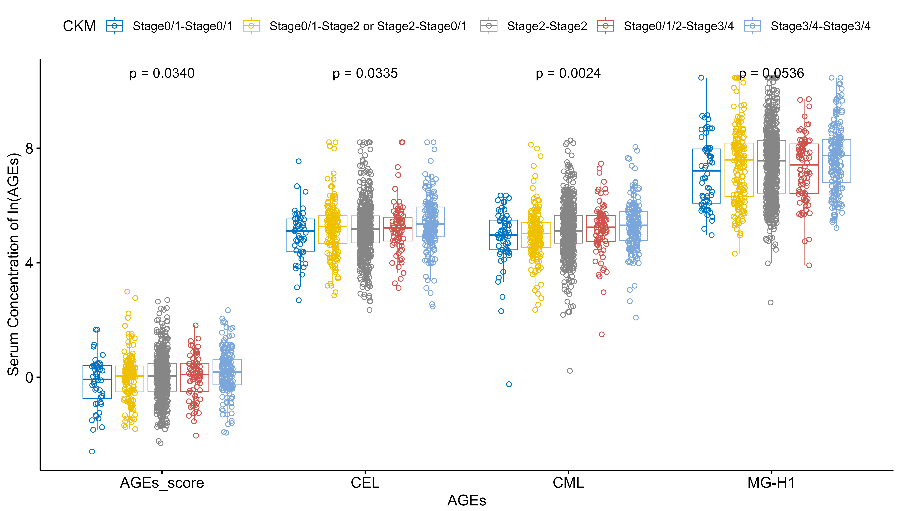 |
| --- | --- | --- |
| 1. 2019-2022   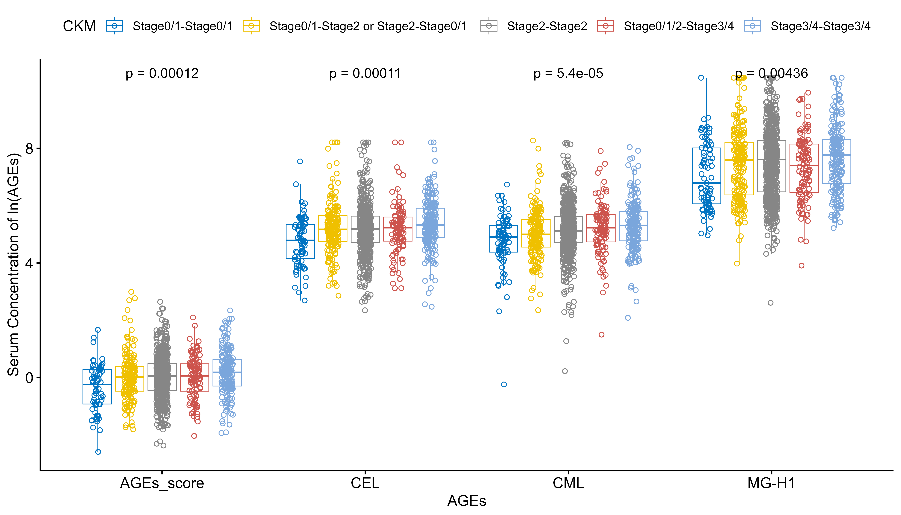 |  |  |

**Figure S4. Differences in AGEs in different transition groups of CKM in different periods**

| 1. AGEs score   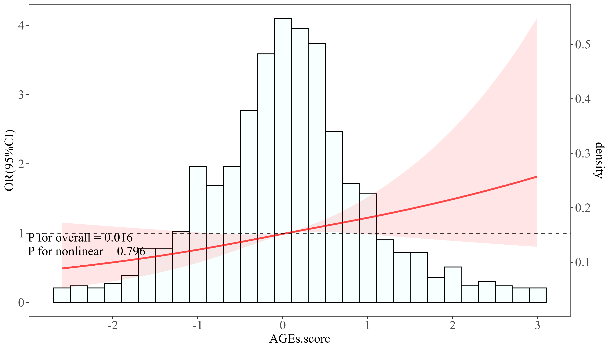 |  | 1. CEL   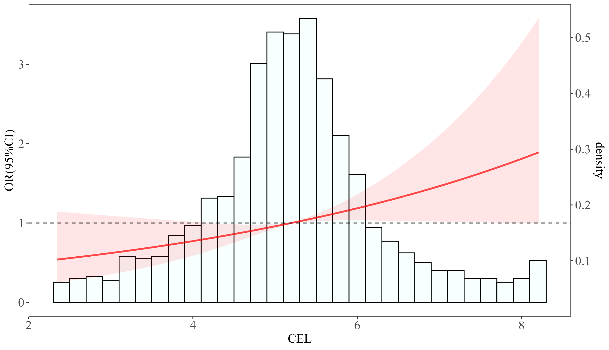 |
| --- | --- | --- |
| 1. CML   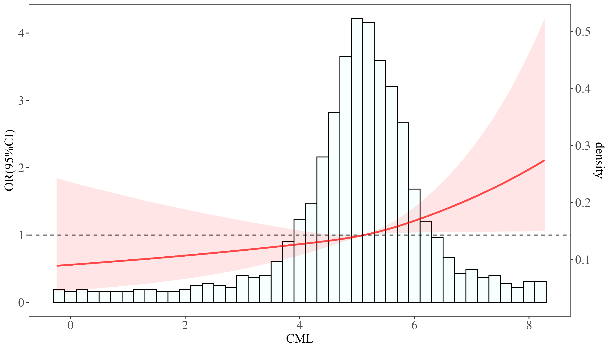 |  | 1. MG-H1   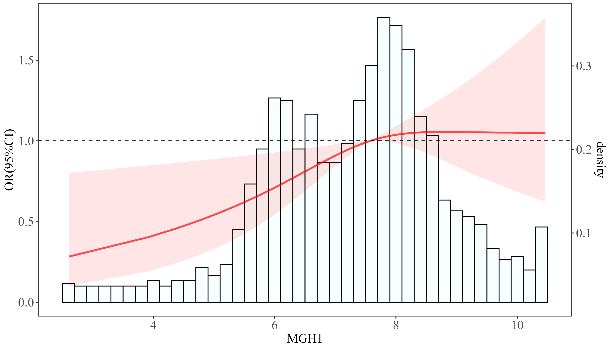 |

**Figure S5. Linear/nonlinear relationship between AGEs score/concentration of specific serum AGEs and different transition groups of CKM in 2019-2022**
