# Supplementary material for: Association Between Serum Advanced Glycation End Products and Cardiovascular‐Kidney‐Metabolic (CKM) Syndrome: A 3‐Year Longitudinal Cohort Study (2019–2022)
Source: J Diabetes. 2025 Aug 21;17(8):e70137. doi: 10.1111/1753-0407.70137 (PMC12370401; doi:10.1111/1753-0407.70137)
Supplement: Supplementary file 2 — Data S2: Suppotimg Information. [file JDB-17-e70137-s002.docx]

**Tables**

| Contents |
| --- |
| Table S1. The definitions of CKM syndrome |
| Table S2. LC-MS/MS experimental parameters setting |
| Table S3. Calibration standard concentration curves for each serum AGEs concentration |
| Table S4. Precision, accuracy, recovery for the determination of AGEs |
| Table S5. Demographics and clinical characteristics of subjects at the baseline (N = 1523) |
| Table S6. Demographic and clinical characteristics of subjects in the CKM stage transition group, 2019-2022 |
| Table S7. Sensitivity analysis for relationship between AGEs scores or specific serum AGEs concentrations and CKM at the baseline by combining CKM's stage0 and stage1, stage 3 and stage 4 |
| Table S8. Sensitivity analysis for relationship between AGEs scores and serum specific AGEs concentration with CKM at the baseline by CKM as continuous variable |

**Table S1. The definitions of CKM syndrome^1^:**

| CKM syndrome stages | Definition |
| --- | --- |
| Stage 0: No CKM risk factors | With normal BMI and waist circumference, normoglycemia, normotension, a normal lipid profile, and no evidence of CKD (eGFR stage G1 or G2 and no self-reported CKD and CVD |
| Stage 1: Excess or dysfunctional adiposity | Individuals with overweight/obesity, abdominal obesity, or dysfunctional adipose tissue, without the presence of other metabolic risk factors or CKD |
| Stage 2: Metabolic risk factors and CKD | Hypertriglyceridemia, hypertension, MetS, diabetes, or CKD (eGFR staging at G3 and above) |
| Stage 3: Subclinical CVD in CKM | Very high-risk CKD (stage G4 or G5 CKD) or high predicted 10-y CVD risk |
| Stage 4: Clinical CVD in CKM | Very high risk of CKD staging (stage G4 or G5 CKD) or self-reported CVD in individuals with overweight/obesity or MetS |

**Table S2. LC-MS/MS experimental parameters setting**

| **AGEs** | ***m/z*** | **CV** | **CE** |
| --- | --- | --- | --- |
| **CML** | 204.9681→84.0214^*^ | 26 | 26 |
| **CML** | 204.9681→130.0736^+^ | 26 | 16 |
| **MG-H1** | 229→70^*^ | 28 | 25 |
| **MG-H1** | 229→114^+^ | 28 | 18 |
| **CEL** | 219.0243→84.0886^*^ | 28 | 34 |
| **CEL** | 219.0243→130.0748^+^ | 28 | 20 |
| **CML-d_4_** | 204.9681→88.0549^*^ | 28 | 28 |
| **CML-d_4_** | 204.9681→134.0939^+^ | 28 | 16 |

Note: Abbreviations: CML, Carboxymethyllysine; CEL, Carboxyethyllysine; MG-H1, Methylglyoxyl-hydroimidazolone-1; *Quantification daughter ion, +Confirmed daughter ion; CV, Cone Voltage; CE, collision energy; LC–MS/MS, liquid chromatography tandem mass spectrometry.

**Table S3. Calibration standard concentration curves for each serum AGEs concentration**

|  | **CML (ppb)** | **CEL (ppb)** | **MG-H1 (ppb)** |
| --- | --- | --- | --- |
| **S1** | 2.5 | 2.5 | 25 |
| **S2** | 5 | 5 | 50 |
| **S3** | 10 | 10 | 100 |
| **S4** | 20 | 20 | 200 |
| **S5** | 50 | 50 | 500 |
| **S6** | 100 | 100 | 1000 |
| **S7** | 200 | 200 | 2000 |
| **S8** | 400 | 400 | 4000 |

Note: Abbreviations: CML, Carboxymethyllysine; CEL, Carboxyethyllysine; MG-H1, Methylglyoxyl-hydroimidazolone-1; ppb, part per billion.

**Table S4. Precision, accuracy, recovery for the determination of AGEs**

| **AGEs** | **Analytes** | **Intra-day** | **Inter-day** | **Recovery（%）** |
| --- | --- | --- | --- | --- |
|  | **concentration (ppb)** | **RSD(%)(n=6)** | **RSD(%)(n=3)** |  |
| **CML** | 10 | 11.07 | 11.57 | --- |
|  | 50 | 7.46 | 5.38 | 72.46 |
|  | 100 | 2.45 | 5.45 | 88.15 |
|  | 400 | --- | --- | 102.17 |
| **CEL** | 10 | 2.64 | 7.28 | --- |
|  | 50 | 3.38 | 1.99 | 55.97 |
|  | 100 | 1.81 | 9.58 | 88.78 |
|  | 400 | --- | --- | 88.22 |
| **MG-H1** | 100 | 4.91 | 15.17 | --- |
|  | 500 | 6.26 | 9.36 | 74.07 |
|  | 1000 | 4.61 | 7.27 | 85.35 |
|  | 4000 | --- | --- | 96.04 |

| **Table S5 Demographics and clinical characteristics of subjects at the baseline (N = 1523)** | | | | | | |
| --- | --- | --- | --- | --- | --- | --- |
| **Characteristic** | **Stage 0 (n=25)** | **Stage 1 (n=188)** | **Stage 2 (n=1082)** | **Stage 3 (n=88)** | **Stage 4 (n=140)** | **P-value** |
| **Age, years** | 43.72±8.57 | 46.55±8.27 | 53.68±9.25 | 64.65±8.34 | 56.72±8.85 | <0.001 |
| **Male, n (%)** | 14 (56.0) | 79 (42.0) | 518 (47.9) | 52 (59.1) | 70 (50.0) | 0.096 |
| **Education** |  |  |  |  |  | 0.020 |
| **Primary school or below** | 19 (76.0) | 128 (68.1) | 754 (69.9) | 76 (86.4) | 99 (70.7) |  |
| **Middle schoo or further** | 6 (24.0) | 60 (31.9) | 324 (30.1) | 12 (13.6) | 41 (29.3) |  |
| **Occasional/Past smoking, n (%)** | 8 (32.0) | 47 (25.3) | 216 (20.1) | 11 (12.5) | 33 (24.1) | 0.060 |
| **Occasional/Past drinking, n (%)** | 4 (16.0) | 6 (3.2) | 31 (2.9) | 3 (3.4) | 9 (6.6) | 0.002 |
| **Occasional/Past tea drinking, n (%)** | 8 (32.0) | 45 (24.1) | 265 (24.5) | 21 (23.9) | 42 (30.0) | 0.595 |
| **Physical activity, n (%)** |  |  |  |  |  | <0.001 |
| **Light** | 14 (58.3) | 126 (68.5) | 706 (66.8) | 76 (87.4) | 104 (74.8) |  |
| **Moderate/Heavy** | 10 (41.7) | 58 (31.5) | 351 (33.2) | 11 (12.6) | 35 (25.2) |  |
| **DII** | -0.14±0.70 | -0.07±0.86 | 0.20±0.96 | 0.47±1.10 | 0.31±1.07 | <0.001 |
| **BMI, kg/m^2^** | 20.52±1.75 | 26.34±3.90 | 27.65±4.31 | 27.74±4.22 | 27.73±4.15 | <0.001 |
| **Waist circumference, cm** | 74.92±6.74 | 86.05±10.36 | 91.91±11.37 | 94.02±11.29 | 94.88±11.77 | <0.001 |
| **Fasting glucose, mmol/L** | 4.96±0.31 | 5.36±0.63 | 7.02±2.74 | 7.93±4.19 | 7.52±2.86 | <0.001 |
| **Triglycerides, mmol/L** | 0.81±0.35 | 0.95±0.31 | 1.74±1.12 | 1.73±1.70 | 1.80±1.64 | <0.001 |
| **HDL‐C, mmol/L** | 1.75±0.35 | 1.49±0.43 | 1.33±0.41 | 1.29±0.38 | 1.39±0.38 | <0.001 |
| **eGFR, mL/min/1.73 m^2^** | 87.98±20.62 | 92.09±17.51 | 83.03±18.88 | 65.84±19.37 | 79.61±19.39 | <0.001 |
| **Diabetes, n (%)** | 0 (0.0) | 0 (0.0) | 479 (44.4) | 62 (70.5) | 66 (47.1) | <0.001 |
| **Hypertension, n (%)** | 0 (0.0) | 0 (0.0) | 842 (77.8) | 83 (94.3) | 109 (77.9) | <0.001 |
| **Hypertriglyceridemia, n (%)** | 0 (0.0) | 0 (0.0) | 432 (39.9) | 30 (34.1) | 57 (40.7) | <0.001 |
| **High-density lipoprotein abnormality, n (%)** | 0 (0.0) | 34 (22.5) | 354 (35.4) | 35 (39.8) | 30 (30.3) | <0.001 |
| **Metabolic syndrome, n (%)** | 0 (0.0) | 0 (0.0) | 737 (68.1) | 66 (75.0) | 92 (65.7) | <0.001 |
| **CML, nmol/L** | 99.11 (41.01, 221.91) | 151.56 (90.54, 231.96) | 164.55 (106.49, 279.04) | 216.03 (135.27, 396.51) | 182.25 (95.85, 311.26) | <0.001 |
| **CEL, nmol/L** | 96.67 (45.96, 205.06) | 164.65 (79.41, 260.63) | 183.53 (115.75, 279.39) | 228.67 (138.94, 413.98) | 198.12 (121.73, 335.84) | <0.001 |
| **MG-H1, nmol/L** | 532.26 (364.24, 1,180.81) | 1,896.91 (534.30, 3,427.85) | 1,981.10 (650.08, 3,713.78) | 2,630.26 (851.01, 5,925.12) | 1,938.35 (860.21, 3,516.77) | <0.001 |
| **AGEs score** | -0.81 (-1.26, -0.17) | -0.02 (-0.69, 0.41) | 0.04 (-0.45, 0.48) | 0.22 (-0.31, 0.93) | 0.15 (-0.28, 0.47) | <0.001 |

Note: Continuous variables were described by Mean ± SD or Median (IQR) and categorical variables were described by n (%). Kruskal Wallis rank sum test was used for statistical inference.

| **Table S6. Demographic and clinical characteristics of subjects in the CKM stage transition group, 2019-2022** | | | | | | |
| --- | --- | --- | --- | --- | --- | --- |
| **Characteristic** | **Stage0/1→Stage0/1 (n=85)** | **Stage0/1→Stage2 or Stage2→Stage0/1  (n=200)** | **Stage2→Stage2 (n=667)** | **Stage0/1/2→Stage3/4 (n=112)** | **Stage3/4→Stage3/4 (n=200)** | ***P*-value** |
| **Age, years** | 44.35±8.39 | 48.08±8.16 | 54.07±8.65 | 59.57±8.71 | 59.79±9.51 | <0.001 |
| **Male, n (%)** | 36 (42.4) | 97 (48.5) | 306 (45.9) | 55 (49.1) | 105 (52.5) | 0.433 |
| **Education** |  |  |  |  |  | 0.007 |
| **Primary school or below** | 54 (63.5) | 124 (62.3) | 476 (71.6) | 80 (71.4) | 156 (78.0) |  |
| **Middle schoo or further** | 31 (36.5) | 75 (37.7) | 189 (28.4) | 32 (28.6) | 44 (22.0) |  |
| **Occasional/Past smoking, n (%)** | 26 (30.6) | 46 (23.2) | 131 (19.7) | 17 (15.5) | 38 (19.3) | 0.079 |
| **Occasional/Past drinking, n (%)** | 3 (3.5) | 4 (2.0) | 23 (3.5) | 3 (2.7) | 12 (6.1) | 0.301 |
| **Occasional/Past tea drinking, n (%)** | 19 (22.6) | 46 (23.0) | 162 (24.3) | 29 (25.9) | 55 (27.5) | 0.829 |
| **Physical activity, n (%)** |  |  |  |  |  | <0.001 |
| **Light** | 58 (69.0) | 117 (60.0) | 450 (69.0) | 89 (79.5) | 162 (81.4) |  |
| **Moderate/Heavy** | 26 (31.0) | 78 (40.0) | 202 (31.0) | 23 (20.5) | 37 (18.6) |  |
| **DII** | -0.05±0.89 | 0.06±0.89 | 0.19±0.96 | 0.32±0.99 | 0.38±1.09 | <0.001 |
| **BMI, kg/m^2^** | 25.93±4.08 | 27.28±4.21 | 27.69±4.24 | 28.49±4.58 | 27.79±4.16 | 0.001 |
| **Waist circumference, cm** | 86.02±11.05 | 88.68±10.95 | 92.05±11.15 | 94.30±11.53 | 94.48±11.77 | <0.001 |
| **Fasting glucose, mmol/L** | 5.30±0.58 | 5.83±1.25 | 7.08±2.88 | 7.84±3.29 | 7.75±3.55 | <0.001 |
| **Triglycerides, mmol/L** | 0.95±0.30 | 1.40±1.09 | 1.73±1.07 | 1.80±1.16 | 1.80±1.74 | <0.001 |
| **HDL‐C, mmol/L** | 1.53±0.41 | 1.40±0.46 | 1.33±0.39 | 1.32±0.40 | 1.34±0.39 | 0.004 |
| **eGFR, mL/min/1.73 m^2^** | 88.91±17.58 | 85.83±21.79 | 83.61±17.96 | 80.69±18.66 | 73.90±20.90 | <0.001 |
| **Diabetes, n (%)** | 0 (0.0) | 37 (18.7) | 297 (44.6) | 65 (58.0) | 113 (56.5) | <0.001 |
| **Hypertension, n (%)** | 0 (0.0) | 71 (35.5) | 543 (81.4) | 91 (81.3) | 169 (84.5) | <0.001 |
| **Hypertriglyceridemia, n (%)** | 0 (0.0) | 46 (23.0) | 269 (40.3) | 53 (47.3) | 78 (39.0) | 0.004 |
| **High-density lipoprotein abnormality, n (%)** | 17 (23.6) | 53 (29.8) | 204 (33.4) | 41 (39.4) | 58 (35.6) | 0.186 |
| **Metabolic syndrome, n (%)** | 0 (0.0) | 69 (34.5) | 467 (70.0) | 90 (80.4) | 137 (68.5) | <0.001 |
| **CML, nmol/L** | 135.11 (79.11, 203.04) | 148.30 (94.90, 250.68) | 167.77 (110.19, 276.56) | 186.85 (113.24, 296.32) | 201.91 (117.96, 328.01) | <0.001 |
| **CEL, nmol/L** | 119.99 (64.06, 208.19) | 176.43 (115.75, 284.28) | 179.06 (111.68, 280.70) | 186.98 (115.29, 272.75) | 205.29 (132.19, 374.96) | <0.001 |
| **MG-H1, umol/L** | 887.39 (419.56, 3,046.49) | 1,975.77 (580.46, 3,739.33) | 2,013.49 (646.36, 3,926.43) | 1,638.83 (633.43, 3,476.80) | 2,360.64 (877.52, 4,079.10) | 0.009 |
| **AGEs score** | -0.24 (-0.92, 0.27) | 0.02 (-0.48, 0.40) | 0.05 (-0.45, 0.48) | 0.06 (-0.47, 0.48) | 0.18 (-0.31, 0.64) | <0.001 |

Note: Continuous variables were described by Mean ± SD or Median (IQR) and categorical variables were described by n (%). Kruskal Wallis rank sum test was used for statistical inference.

| **Table S7. Sensitivity analysis for relationship between AGEs scores or specific serum AGEs concentrations and CKM at the baseline by combining CKM's stage0 and stage1, stage 3 and stage 4** | | | | | | |  |
| --- | --- | --- | --- | --- | --- | --- | --- |
| **CKM stage** | | **Q1** | **Q2** | **Q3** | **Q4** | **P- trend** | **1-SD increment** |
|  |  | **[-2.6,-0.45]** | **(-0.45,0.0428]** | **(0.0428,0.484]** | **(0.484,2.99]** |  |  |
| **CKM stage 0&1 vs. 2 vs. 3&4, n** | |  |  |  |  |  |  |
| **Serum AGEs score levels** | |  |  |  |  |  |  |
| **Model1** | | 1.00 (ref.) | 1.80(1.25, 2.58) | 1.96(1.33, 2.89) | 2.42(1.67, 3.52) | <0.001 | 1.43(1.22, 1.68) |
| **Model2** | | 1.00 (ref.) | 1.71(1.18, 2.49) | 1.62(1.09, 2.41) | 1.95(1.33, 2.86) | <0.001 | 1.30(1.10, 1.54) |
| **Serum CEL levels** | |  |  |  |  |  |  |
| **Model1** | | 1.00 (ref.) | 1.70(1.20, 2.41) | 1.41(0.99, 2.02) | 2.31(1.63, 3.28) | <0.001 | 1.31(1.15, 1.51) |
| **Model2** | | 1.00 (ref.) | 1.67(1.17, 2.39) | 1.21(0.84, 1.75) | 2.02(1.41, 2.90) | 0.001 | 1.25(1.09, 1.44) |
| **Serum CML levels** | |  |  |  |  |  |  |
| **Model1** | | 1.00 (ref.) | 1.14(0.83, 1.58) | 1.33(0.96, 1.84) | 1.95(1.41, 2.69) | <0.001 | 1.33(1.17, 1.50) |
| **Model2** | | 1.00 (ref.) | 1.06(0.77, 1.48) | 1.16(0.83, 1.62 ) | 1.46(1.05, 2.04) | 0.022 | 1.19(1.05, 1.36) |
| **Serum MG-H1 levels** | |  |  |  |  |  |  |
| **Model1** | | 1.00 (ref.) | 1.74(1.25, 2.41) | 1.96(1.32, 2.90) | 2.20(1.49, 3.26) | <0.001 | 1.25(1.11, 1.40) |
| **Model2** | | 1.00 (ref.) | 1.66(1.18, 2.33) | 1.64(1.09, 2.47) | 1.83(1.22, 2.75 ) | 0.007 | 1.17(1.04, 1.32) |

Note: Ordinal logistic regression was used to analyze the relationship between the different stages of CKM and the quartiles of AGEs, and the strength of association was demonstrated by OR (95% CIs). OR odds ratio, CI confidence interval. Model 1: Only AGEs score or concentration of specific serum AGEs. Model 2: adjusted for age and physical activity. P-trend: Linear trend test using the median value of each category. Q1 Quartile 1, Q2 Quartile 2, Q3 Quartile 3 and Q4 Quartile 4.

| **Table S8. Sensitivity analysis for relationship between AGEs scores and serum specific AGEs concentration with CKM at the baseline by CKM as continuous variable** | | | | | | |
| --- | --- | --- | --- | --- | --- | --- |
| **CKM (continuous variable)** | **Q1** | **Q2** | **Q3** | **Q4** | **P-trend** | **1-SD increment** |
|  | **[-2.6,-0.45]** | **(-0.45,0.0428]** | **(0.0428,0.484]** | **(0.484,2.99]** |  |  |
| **Serum AGEs score** |  |  |  |  |  |  |
| **Model1** | ref. | 0.22(0.09, 0.35) | 0.28(0.14, 0.42) | 0.30(0.17, 0.44) | <0.001 | 0.12(0.06, 0.18) |
| **Model2** | ref. | 0.17(0.04, 0.29) | 0.19(0.05, 0.32) | 0.20(0.07, 0.33) | 0.004 | 0.08(0.02, 0.13) |
| **Serum CEL levels** |  |  |  |  |  |  |
| **Model1** | ref. | 0.18(0.06, 0.31) | 0.12(-0.01, 0.25) | 0.28(0.16, 0.41) | <0.001 | 0.09(0.04, 0.14) |
| **Model2** | ref. | 0.15(0.03, 0.27) | 0.03(-0.09, 0.16) | 0.21(0.09, 0.33) | 0.004 | 0.07(0.02, 0.11) |
| **Serum CML levels** |  |  |  |  |  |  |
| **Model1** | ref. | 0.02(-0.10, 0.13) | 0.08(-0.04, 0.19) | 0.21(0.09, 0.33) | <0.001 | 0.09(0.05, 0.14) |
| **Model2** | ref. | -0.02(-0.14, 0.09) | 0.02(-0.09, 0.14 ) | 0.10(-0.01, 0.22) | 0.049 | 0.05(0.01, 0.09) |
| **Serum MG-H1 levels** |  |  |  |  |  |  |
| **Model1** | ref. | 0.23(0.12, 0.35) | 0.27(0.13, 0.41) | 0.29(0.15, 0.42) | <0.001 | 0.07(0.03, 0.12) |
| **Model2** | ref. | 0.18(0.07, 0.29) | 0.18(0.04, 0.32) | 0.18(0.04, 0.32) | 0.018 | 0.04(0.00, 0.08) |

Note: Multiple linear regression was used to analyze the relationship between the different stages of CKM and the quartiles of AGEs, demonstrating the strength of association using beta (95% CIs). beta regression coefficient, CI confidence interval. Model 1 Only AGEs score or concentration of specific serum AGEs. Model 2: adjusted for age, educational level, alcohol consumption and physical activity. P-trend: Linear trend test using the median value of each category. Q1 Quartile 1, Q2 Quartile 2, Q3 Quartile 3 and Q4 Quartile 4.
